# Quantitative Analysis of the Cytoskeleton’s Role in Inward Rectifier K_*IR*_2.1 Forward and Backward Trafficking

**DOI:** 10.3389/fphys.2021.812572

**Published:** 2022-01-25

**Authors:** Encan Li, Vera Loen, Willem B. van Ham, Willy Kool, Marcel A. G. van der Heyden, Hiroki Takanari

**Affiliations:** ^1^Department of Medical Physiology, Division of Heart & Lungs, University Medical Center Utrecht, Utrecht, Netherlands; ^2^Department of Interdisciplinary Researches for Medicine and Photonics, Institute of Post-LED Photonics, University of Tokushima, Tokushima, Japan

**Keywords:** ion channel, trafficking, cytoskeleton, Chloroquine, Nocodazole, Cytochalasin, Dendra2, patch clamp

## Abstract

Alteration of the inward rectifier current *I*_*K*1_, carried by K_IR_2.1 channels, affects action potential duration, impacts resting membrane stability and associates with cardiac arrhythmias. Congenital and acquired K_IR_2.1 malfunction frequently associates with aberrant ion channel trafficking. Cellular processes underlying trafficking are intertwined with cytoskeletal function. The extent to which the cytoskeleton is involved in K_IR_2.1 trafficking processes is unknown. We aimed to quantify the dependence of K_IR_2.1 trafficking on cytoskeleton function. GFP or photoconvertible Dendra2 tagged K_IR_2.1 constructs were transfected in HEK293 or HeLa cells. Photoconversion of the Dendra2 probe at the plasma membrane and subsequent live imaging of trafficking processes was performed by confocal laser-scanning microscopy. Time constant of green fluorescent recovery (τg,s) represented recruitment of new K_IR_2.1 at the plasma membrane. Red fluorescent decay (τr,s) represented internalization of photoconverted K_IR_2.1. Patch clamp electrophysiology was used to quantify *I*_*KIR*2_._1_. Biochemical methods were used for cytoskeleton isolation and detection of K_IR_2.1-cytoskeleton interactions. Cytochalasin B (20 μM), Nocodazole (30 μM) and Dyngo-4a (10 nM) were used to modify the cytoskeleton. Chloroquine (10 μM, 24 h) was used to impair K_IR_2.1 breakdown. Cytochalasin B and Nocodazole, inhibitors of actin and tubulin filament formation respectively, strongly inhibited the recovery of green fluorescence at the plasma membrane suggestive for inhibition of K_IR_2.1 forward trafficking [τg,s 13 ± 2 *vs.* 131 ± 31* and 160 ± 40* min, for control, Cytochalasin B and Nocodazole, respectively (*p < 0.05 *vs.* control)]. Dyngo-4a, an inhibitor of dynamin motor proteins, strongly slowed the rate of photoconverted channel internalization, whereas Nocodazole and Cytochalasin B had less effect [τr,s 20 ± 2 *vs.* 87 ± 14*, 60 ± 16 and 64 ± 20 min (*p < 0.05 *vs.* control)]. Cytochalasin B treatment (20 μM, 24 h) inhibited *I*_*KIR*2_._1_. Chloroquine treatment (10 μM, 24 h) induced intracellular aggregation of K_IR_2.1 channels and enhanced interaction with the actin/intermediate filament system (103 ± 90 fold; *p* < 0.05 *vs.* control). Functional actin and tubulin cytoskeleton systems are essential for forward trafficking of K_IR_2.1 channels, whereas initial backward trafficking relies on a functional dynamin system. Chronic disturbance of the actin system inhibits K_IR_2.1 currents. Internalized K_IR_2.1 channels become recruited to the cytoskeleton, presumably in lysosomes.

## Introduction

Inward rectifier potassium channels from the K_IR_2.x protein family are present in many different excitable cell types. Ventricular cardiomyocytes strongly express K_IR_2.1 and K_IR_2.2 channel proteins (De Boer et al., 2010). Homo- or heterotetrameric K_IR_2.x assemblies form the functional channels that underlie the inward rectifier current (*I*_*K*1_) (Hibino et al., 2010). *I*_*K*1_ contributes to the final repolarization phase of the cardiac action potential and stabilizes the resting membrane potential of these cells (Van der Heyden and Jespersen, 2016). *I*_*K*1_ loss-of-function, either genetic or pharmacological, can result in cardiac action potential prolongation and ectopic activity in both model systems and patients (Biliczki et al., 2002; Tristani-Firouzi et al., 2002; Takanari et al., 2013; Myles et al., 2015; Reilly et al., 2020). In contrast, *I*_*K*1_ gain-of-function associates with action potential shortening and reentry-based arrhythmia mechanisms, as for example atrial fibrillation (Xia et al., 2005; Kharche et al., 2008; Hattori et al., 2012; Deo et al., 2013).

K_IR_2.1 proteins possess a short N-terminal and long C-terminal intracellular domain, two transmembrane domains, and two very short extracellular domains divided by the transmembrane pore domain. The intracellular domains contain several specific highly conserved sequences for intracellular transport, i.e., endoplasmic reticulum and Golgi export sequences, and sequences for subcellular anchoring and protein-protein interaction, such as the C-terminal PDZ domain (Stockklausner et al., 2001; Sampson et al., 2003; Stockklausner and Klocker, 2003; Leonoudakis et al., 2004a,b; Hofherr et al., 2005; Ma et al., 2011; Houtman et al., 2014; Li et al., 2016). Proper expression of the necessary amount of K_IR_2.1 proteins at the plasma membrane is of crucial importance for normal channel function in the context of an action potential (Willis et al., 2015). The cytoskeleton has an important role in processes that transport K_IR_2.1, and other cardiac ion channels, to and from the plasma membrane, known as forward and backward trafficking, and in anchoring K_IR_2.1 at the plasma membrane (Vatta and Faulkner, 2006; Steele and Fedida, 2014). Importantly, many loss-of-function mutations in the K_IR_2.1 gene associated with clinical phenotypes result in aberrant trafficking (Zangerl-Plessl et al., 2019).

The cytoskeleton consists of four types of filaments (Mostowy and Cossart, 2012). Actin filaments are 7 nm diameter, double stranded, helical assemblies of α, β or γ-type ± 42 kDa globular actin monomers. Tubulin filaments are 23 nm diameter hollow cylinders formed by α/β-tubulin (±50 kDa) dimers. A number of different intermediate filaments (11 nm diameter) proteins exist that may display cell type specific expression, such as vimentin in mesenchymal and keratin proteins in epithelial cells. Finally, septin proteins (30–66 kDa) form complexes that can organize as filaments which can further assemble as bundles and even circular structures. Actin and tubulin filaments are mainly involved in trafficking processes of ion channels (Steele and Fedida, 2014). Specific motor proteins interact with ion channels and transport their cargo along the filament systems. For example, K_IR_2.1 internalization depends on functional dynamin motor proteins, as their inhibition by dynasore or overexpression of the dynactin p50, increases expression at the plasma membrane (Loewen et al., 2009; Varkevisser et al., 2013). Pharmacological disruption of the actin and tubulin filament systems can be achieved experimentally by the use of Cytochalasin B and Nocodazole, respectively (De Brabander et al., 1976; MacLean-Fletcher and Pollard, 1980). Remarkably, in contrast to several other potassium channels whose expression at the plasma membrane increases upon Nocodazole mediated disruption of the tubulin system, K_IR_2.1 expression was found to decrease (Loewen et al., 2009). The authors suggest that substantial tubulin-dependent forward trafficking might explain this phenomenon. Currently, however, quantitative data on the roles of the actin and tubulin on K_IR_2.1 forward and backward trafficking are not available.

We demonstrated previously that the antimalarial drug Chloroquine (CQ) increased K_IR_2.1 expression and *I*_*K*1_. CQ also induced intracellular accumulation of K_IR_2.1-GFP and led to the presence of an N-terminal cleavage product (Jansen et al., 2008; Varkevisser et al., 2013). The latter two phenomena most likely result from direct or indirect alkalization of the lysosome by CQ affecting its function (Ohkuma and Poole, 1978; Ponsford et al., 2021). In kidney cells, CQ has also been shown to affect the cytoskeleton (Kang et al., 2020). To which extent this affects interaction of K_IR_2.1 with the cytoskeleton is unknown.

In the current work we made use of fluorescently C-terminally tagged K_IR_2.1 proteins (GFP and Dendra2), which are amendable for fluorescent microscopy and western blot analysis, and do not interfere with electrophysiological properties (De Boer et al., 2006b; Korte et al., 2017). The Dendra2 tag can be irreversibly photoconverted from green to red fluorescence by short UV illumination (Chudakov et al., 2007). In real-time microscopy this allows visualization of trafficking effects of specific pools of the K_IR_2.1-Dendra2 protein. By use of this technology, we determined alterations in K_IR_2.1 forward and backward trafficking kinetics in response to cytoskeleton-modifying compounds. Furthermore, we assessed the interaction of K_IR_2.1 with the cytoskeleton and the effects of Chloroquine treatment on these.

## Materials and Methods

### Cell Culture

HeLa cells were cultured in low glucose Dulbeco’s Modified Eagle Medium supplemented with 10% fetal calf serum and 1% penicillin/streptomycin, all purchased from Wako (Osaka, Japan). Human Embryonal Kidney 293 cells stably expressing K_IR_2.1 channel tagged with GFP [HEK-KWGF (De Boer et al., 2006b)] and Chinese Hamster Ovary cells stably expressing K_IR_2.1 channel tagged with Dendra2 [CHO-Dendra2 (Korte et al., 2017)] were cultured in the same medium, but purchased from Lonza (Walkersville, MD, United States). In time course experiments, cells were seeded and harvested on identical days.

### Drugs

We purchased Dyngo-4a and Nocodazole from Abcam (Cambridge, United Kingdom). Cytochalasin B was purchased from Sigma Aldrich Japan (Kawasaki, Japan). The chemicals were dissolved in DMSO to provide a stock solution of 10 mM. All compounds were aliquoted and stored at −20°C until use. Cells were exposed to the drugs by adding stock solutions to the cell medium at the following concentrations: Dyngo-4a, 10 nM; Nocodazole, 30 μM; Cytochalasin B, 20 μM, Chloroquine, 10 μM.

### Western Blot

Cell lysates were prepared in buffer D [20 mM HEPES, 125 mM NaCl, 10% glycerol, 1 mM EGTA, 1 mM dithiothreitol, 1 mM EDTA, and 1% Triton X-100 (pH 7.6)] supplemented with 1 mM phenylmethylsulfonyl fluoride (PMSF) and 10 μg⋅mL^–1^ aprotinin. Lysates were clarified by centrifugation at 14,000 rpm for 10 min at 4°C. Protein concentrations were established by BCA Protein Assay (Thermo Fisher Scientific, Breda, Netherlands). Samples were added to loading buffer and 25 μg of proteins were separated by 7.5% SDS–PAGE and blotted on a nitrocellulose membrane (Bio-Rad Laboratories, Veenendaal, Netherlands). Ponceau S staining was used for subsequent quantification. Blots were blocked with 5% Protifar dissolved in Tris-buffered saline/Tween 20 [20 mM Tris-HCl (pH 8.0), 150 mM NaCl, 0.05% Tween-20 (v/v)] for 1 h at room temperature. Blots were incubated with Dendra2 (Origene, Rockville, MD, United States) primary antibody and peroxidase-conjugated secondary antibody (Jackson ImmunoResearch, West Grove, PA, United States). Standard ECL procedure was used for final detection (Amersham Bioscience, Buckinghamshire, United Kingdom). Quantification was done by Image Lab software version 6.1 (Bio-Rad Laboratories, Veenendaal, Netherlands).

### Live Imaging With Confocal Microscopy

HeLa cells were cultured on 10% poly-L-lysine-coated small glass-bottom dishes (Iwaki, Shizuoka, Japan). Cells were transfected with the K_IR_2.1-Dendra2 construct using Lipofectamine 2000 (Thermo Fisher Scientific, Yokohama, Japan). Twenty-four hours after transfection, the medium of the transfected dishes was replaced by fresh supplemented DMEM. At least 1 h after changing the medium, dishes were treated with various drugs when not used for baseline measurements. In case of baseline measurements, the DMEM of the dishes was changed just before imaging to DMEM without phenol red supplemented with 10% fetal calf serum, 1% penicillin/streptomycin and 25 mM HEPES buffer (Dojin, Kyoto, Japan). In case of measurements of treated cells, dishes were treated 24 h prior to imaging. For treatment the DMEM of the dishes was changed to DMEM without phenol red supplemented with 10% fetal calf serum, 1% penicillin/streptomycin, 25 mM HEPES buffer and either 10 nM Dyngo-4a, 30 μM Nocodazole or 20 μM Cytochalasin B. Imaging was carried out using a Nikon A1R confocal microscope (Nikon, Tokyo, Japan) with a 60x oil immersion objective. The cells were maintained using a stage-top incubator providing a 37°C, 5% CO_2_, humid environment (TOKAI HIT, Fujinomiya, Japan). Photoconversion of K_IR_2.1-Dendra2 was carried out on a small region of interest (ROI) by irradiation of a blue laser (405 nm) at 6% power for 1 s. To prevent substantial photobleaching, the specific recommendations of Chudakov et al. (2007) with respect to Dendra2 were applied. Green signal was excited by a 488-nm laser with 1.5% power and emitted light was obtained through a FITC band-path filter (535/45 nm). Red signal was excited by a 562-nm laser with 3% power and fluorescence was recorded through a TRITC band-path filter (605/55 nm). In total, following photoconversion 26 images were taken for each channel. Data were processed using NIS Elements software (Nikon, Tokyo, Japan). Fluorescence data for the red and green signal in each (partially) converted cell was divided by its maximum value to make all cells intercomparable. Values for green and red fluorescence intensity were plotted against time (s) and were fitted by the following double exponential equation.


f(x)=f+0A(1-e)-τ1*x1+A(1-e)-τ2*x2

Then the value τ_1_ and τ_2_ for each data series was estimated as time constant for fast and slow component, respectively, to indicate the speed of increase in green fluorescence or decay in red fluorescence. Double exponential fitting was performed using Origin version 8 (Microcal software, Northampton, MA, United States).

### Patch Clamp Electrophysiology

HEK-KWGF cells were grown on 0.1% gelatin (Bio-Rad, Veenendaal, Netherlands) coated Ø 12-mm cover slips in a 24-well plate. Cells were randomly divided into two groups; the treatment groups were treated with 20 μM Cytochalasin B for 24 h, while the control group did not undergo any treatment. *I*_*KIR*2_._1_ from single cells was recorded in whole-cell voltage clamp mode using an Axopatch 200B amplifier (Axon Instruments, Union City, CA, United States) controlled by Clamp10.4 software (Molecular Devices, LLC., San Jose, CA, United States) at room temperature. Voltage clamp measurements were performed by applying 1s test pulses ranging between −120 and +30 mV, in 10 mV increments. Signals were low-pass filtered at 2 kHz and sampled at 4 kHz from a holding potential of −40 mV, with series resistance compensation of at least 70%. Liquid junction potential (LJP) was determined with the built in “Junction Potential Calculator” application of pCLAMP. Using the current solutions, LJP was 13.8 mV. Steady-state current at the end of the pulse was normalized to cell capacitance and plotted versus test potential (corrected for LJP). Patch pipettes were made with a Sutter P-2000 puller (HEKA Elektronik, Lambrecht, Germany) and had resistances of 2–3 MΩ. Extracellular solution for whole cell *I*_*KIR*2_._1_ measurements contained (in mmol/L): NaCl 140, KCl 5.4, CaCl_2_ 1, MgCl_2_ 1, glucose 6, NaHCO_3_ 17.5, HEPES 15, pH 7.4/NaOH. Pipette solution contained potassium gluconate 125, KCl 10, HEPES 5, EGTA 5, MgCl_2_ 2, CaCl_2_ 0.6, Na_2_ATP 4, pH 7.20/KOH.

### Cell Fractionation

Fractionation was performed essentially as described earlier (Van Bergen en Henegouwen et al., 1992). In short, cells were cultured on two 100 mm Ø petri dishes per treatment, until 90% confluency was reached. Then, cells were washed twice with cold PBS. One petri dish was lysed with buffer D, scraped and transferred into a precooled tube. Lysate was spun at 14,000 rpm at 4°C for 5 min (supernatant = whole cell fraction, WC). The second petri dish was lysed with cytoskeleton buffer (10 mM PIPES, 250 mM sucrose, 3 mM MgCl_2_, 150 mM KCl and 1 mM EGTA) with 1% (v/v) Triton X-100 (CSK-100), scraped and transferred into a precooled tube. Lysate was spun at 14,000 rpm at 4°C for 10 min (supernatant = membrane and cytosol fraction, CM). The pellet was resuspended in CSK-100 and spun at 14,000 rpm at 4°C for 10 min. Supernatant was removed and the pellet was resuspended in RIPA lysis buffer (20 mM Tris, 150 mM NaCl, 10 mM Na_2_HPO_4_, 1% (v/v) Triton X-100, 1% (w/v) Na-deoxycholaat, 0.1% (w/v) SDS, 1 mM EDTA and 50 mM NaF) supplemented with 200 mM PMSF and 10 μg/ml aprotinin and spun down at 14,000 rpm at 4°C for 10 min (supernatant = cytoskeleton fraction, CSK).

### Immunofluorescence Microscopy of Fixed Cells

HEK-KWGF cells were cultured on 15 mm Ø 0.1% gelatin coated coverslips. Cells were washed with PBS^++^ (PBS supplemented with 1 mM MgCl_2_ and 1 mM CaCl_2_) and either stayed in PBS^++^ or were incubated two times 5 min with cold CSK-100 buffer at room temperature to isolate the cytoskeleton. Cells were then fixed with ice-cold methanol at −20°C for 20 min. Cells were quenched with 50 mM glycine in PBS two times for 10 min, and blocked with NET-gel (50 mM Tris-HCl, pH 8.0, 150 mM NaCl, 1 mM EDTA, 0.2% (w/v) gelatin, 0.05% Igepal and 0.02% NaN_3_) two times for 15 min. Coverslips were incubated overnight with primary antibody (β-actin, Sigma-Aldrich cat. no. A5444) in NET-gel at room temperature. K_IR_2.1-GFP was imaged using the GFP fluorophore. Secondary antibody (Jackson Laboratories) incubation was done in NET-gel for 120 min at room temperature. Images were made with a Nikon eclipse 80i light microscope with a 60x oil immersion lens and NIS elements software (Nikon Europe B.V., Amsterdam, Netherlands).

### Statistics

Data are expressed as mean ± SD. Differences between group averages were tested using a one-way ANOVA with a *post hoc* test (Tukey’s HSD), or unpaired *T*-test and considered significant when the *p*-value was less than 0.05. Statistical analysis was performed using either GraphPad Prism version 9 (GraphPad software, San Diego, CA, United States) or Origin version 8 (Microcal software, Northampton, MA, United States).

## Results

### K_IR_2.1 Forward Trafficking Depends Strongly on Intact Actin and Tubulin Networks

**FIGURE 1:**
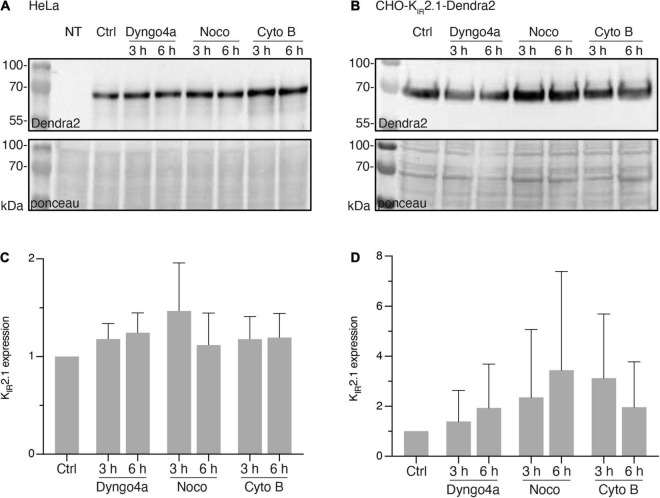
Nocodazole, Cytochalasin B or Dyngo-4a treatment up to 6 h do not affect K_IR_2.1-Dendra2 protein expression level. **(A)** Western blot analysis of K_IR_2.1-Dendra2 expression in HeLa Cells treated for different time period with Dyngo-4a (10 nM), Nocodazole (30 μM) or Cytochalasin B (20 μM) (*n* = 5). Non-transfected cells (NT) were used as negative control. **(B)** Western blot analysis of K_IR_2.1-Dendra2 expression in CHO cells (*n* = 6). **(C)** Summarized results of K_IR_2.1-Dendra2 expression in control and HeLa cells treated for 3 h or 6 h (*n* = 5). Data are presented as the fold of control. **(D)** Summarized results of **(B)**. Data are showed as mean ± SD, control protein level was designed as 100% after correction. Ponceau staining was used as a loading control.

Since interpretation of local subcellular signals obtained by fluorescent microscopy might be hindered by rapid changes in overall expression levels of the fluorescent protein, we first determined expression levels of K_IR_2.1-Dendra2 in response to Dyngo-4a, Nocodazole and Cytochalasin B for up to 6 h by western blot analysis. In transiently transfected HeLa cells (Figure 1A) and stable CHO-K_IR_2.1-Dendra2 cells (Figure 1B), Dyngo-4a, Nocodazole or Cytochalasin B did not significantly alter K_IR_2.1-Dendra2 expression levels for up to 6 h (Figures 1C,D). Therefore, within the intended fluorescent imaging time frame of 1 h (next section), no effects of treatment on overall K_IR_2.1-Dendra2 expression levels were expected.

**FIGURE 2:**
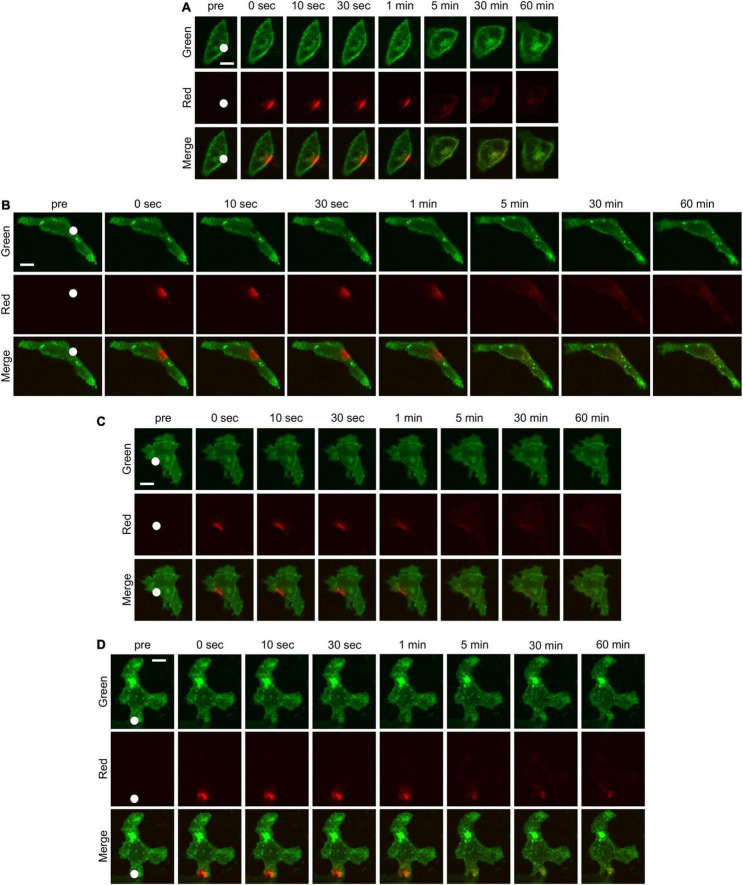
Time-lapse confocal microscopic images of HeLa cells transfected with K_IR_2.1-Dendra2 before and after photoconversion. The cells without any treatment **(A)** and the cells treated with Dyngo-4a **(B)**, Nocodazole **(C)**, Cytochalasin B **(D)** were imaged. The green fluorescence shows the localization of K_IR_2.1 channel before photoconversion and the red fluorescence shows the localization of K_IR_2.1 channel after photoconversion. White circle in each figure indicates the region where a blue laser was irradiated for photoconversion of Dendra2. Scale bar in each images indicate 10 μm.

**FIGURE 3:**
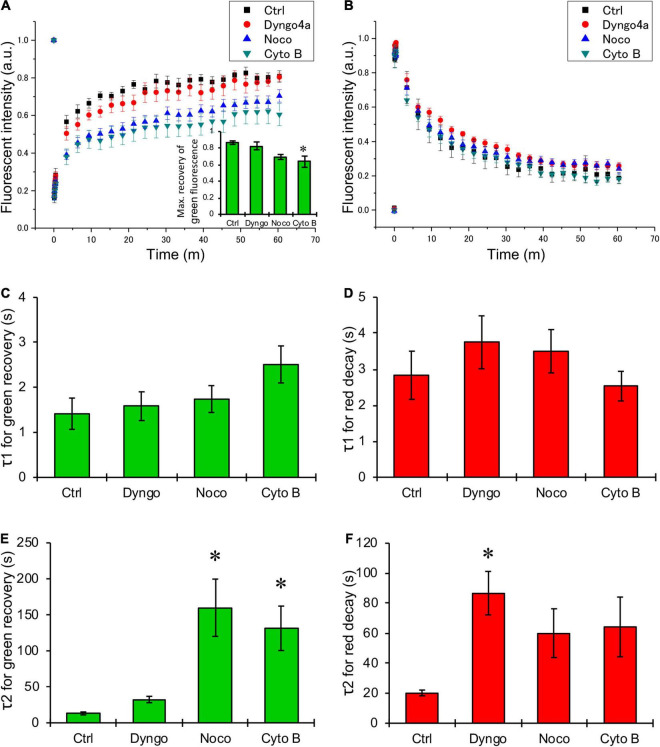
Quantification of changes in green and red fluorescence of K_IR_2.1-Dendra2. **(A,B)** Summarized data of time-dependent recovery in green fluorescence **(A)** and decay of red fluorescence **(B)**. Black boxes are for control (*n* = 14), and red circles, blue triangles, green triangles are for the data from the cells treated with Dyngo-4a (*n* = 15), Nocodazole (*n* = 20), Cytochalasin B (*n* = 17), respectively. **(C,D)** Summarized data of τ1 **(C)** and τ2 **(D)**, fast and slow component of time constant for the recovery of green fluorescence after photoconversion, respectively. **(E,F)** Summarized data of τ1 **(E)** and τ2 **(F)**, fast and slow component of time constant for the decay of red fluorescence after photoconversion, respectively. (**A**, inset) The maximum recovery rate of green fluorescence after photoconversion. *: *p* < 0.05 *vs.* control (one-way ANOVA).

Next, HeLa cells were transiently transfected with K_IR_2.1-Dendra2 to assess forward and backward trafficking of K_IR_2.1 channel. Upon proper plasma membrane expression, local laser-assisted Dendra2 green-to-red conversion was performed using a confocal microscope. Recovery of the green signal (planar movement and forward trafficking) and decay of the red signal (planar movement and backward trafficking) were assessed for up to 60 min. Figure 2 shows representative time-lapse images of HeLa cells transfected with K_IR_2.1-Dendra before and after photoconversion. White circles indicate the region irradiated by the blue laser for photoconversion of Dendra2. Green fluorescence decreased and red fluorescence increased at the region of photoconversion just after blue laser irradiation, followed by time-dependent recovery of green fluorescence and reduction of red fluorescence. Although the shape and the size of the cells were affected by each chemical treatment, the trend of fluorescent changes was the same in all groups. Among them, the decay of red fluorescence appeared to be slower in the cells treated with Dyngo-4a and the recovery of green fluorescence was slower in the cells treated with Nocodazole and Cytochalasin B. Summarized data of green and red fluorescent intensity also revealed that Nocodazole and Cytochalasin B slowed down the recovery of the green signal (blue and green triangles, respectively, in Figure 3A), while Dyngo-4a prolonged the decay of the red signal (red circles in Figure 3B). The maximum recovery rate of green fluorescence at 1 h after photoconversion seemed to be reduced by Nocodazole and Cytochalasin B, especially the treatment with Cytochalasin B resulted in significant decrease in the maximum recovery rate by approximately 40% (inset Figure 3A). Recovery and decay of each fluorescence by time could be best described with a double exponential fitting resulting in two time constants, fast (τ1) and slow (τ2) component, respectively. In non-treated cells, green and red signals displayed a fast time constant (1.4 ± 1.3 min *n* = 14 and 2.8 ± 2.3 min *n* = 12, respectively) presumed as being planar movements. No significant changes in the fast components were observed with Dyngo-4a, Nocodazole or Cytochalasin B treatment (Figures 3C,D). In non-treated cells, τ2 for green recovery and red decay were approximately 8–10 times higher (13.1 ± 7.7 min, *n* = 14 and 20.2 ± 6.7 min, *n* = 12, respectively) than the fast component, and presumably reflect forward and backward trafficking of K_IR_2.1-Dendra2 channels, respectively. Slow recovery of green signal, forward trafficking, was strongly affected by Nocodazole (160.0 ± 177 min, *P* < 0.05, *n* = 20, 12-fold) and Cytochalasin B (131.5 ± 119 min, *P* < 0.05 *n* = 15, 10-fold) (Figure 3E). Dyngo-4a significantly increased τ2 for the red decay, i.e., slowing backward trafficking, (86.7 ± 56 min, *P* < 0.05, *n* = 15) (Figure 3F). Apparent increases in τ2 for red decay by Nocodazole (60.1 ± 72.4 min, *n* = 20) and Cytochalasin B (64.3 ± 81.2 min, *n* = 17) did not reach statistical significance. As impaired forward trafficking might result in intracellular K_IR_2.1-Dendra2 accumulation, we assessed vesicular accumulation (<10 μm structures) and massive accumulation (intracellular structures >10 μm). Indeed, disruption of the cytoskeleton by Nocodazole or Cytochalasin B increased the ratio of cells displaying intracellular accumulations (Supplementary Figure 1).

**FIGURE 4:**
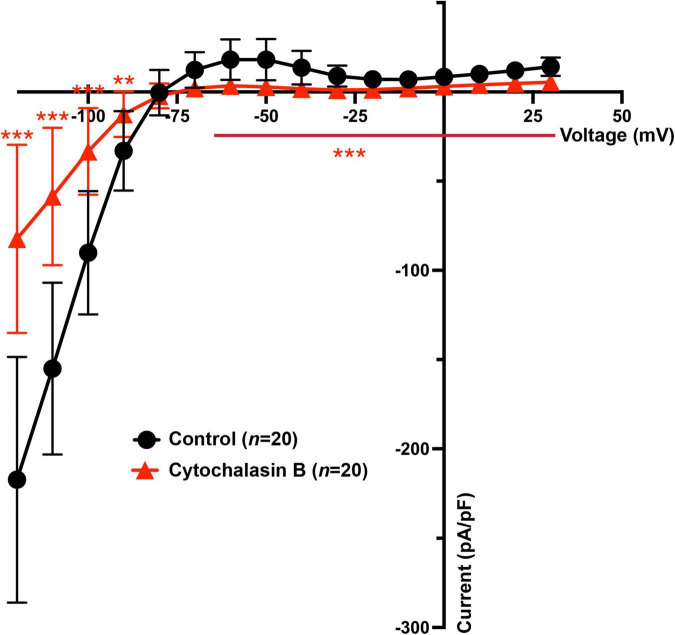
Cytochalasin B treatment decreases both inward and outward *I*_*KIR*2_._1_ in HEK-KWGF cells. Current-voltage relationship of mean *I*_*KIR*2_._1_ current values ± SD. In the treatment group, cells were treated with Cytochalasin B for 24 h with a final concentration of 20 μM. The control group did not undergo any treatment. *n* = 20 cells for each group. **p < 0.01, ***p < 0.001.

Previous and the above results demonstrate that Cytochalasin B and Nocodazole (Loewen et al., 2009) strongly inhibit K_IR_2.1 forward trafficking over backward trafficking, which would result in decreased *I*_*KIR*2_._1_. Therefore, *I*_*KIR*2_._1_ current generated by K_IR_2.1 channels in HEK-KWGF cells was determined following 24 h application of Cytochalasin B by whole-cell patch clamp. It was found that both the inward (from −120 to −90 mV) and outward (−70 to + 30 mV) *I*_*KIR*2_._1_ components were significantly decreased when compared with non-treated HEK-KWGF cells (Figure 4). Similar results were found previously for Nocodazole (Loewen et al., 2009).

### Chloroquine Induces K_IR_2.1-Cytoskeleton Interaction

**FIGURE 5:**
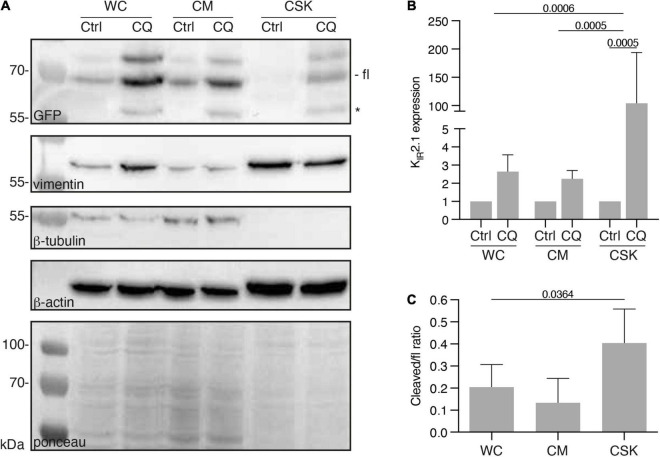
Presence of K_IR_2.1-GFP in the cytoskeleton containing cell fraction, induced by Chloroquine treatment. **(A)** Western blot analysis of whole cell lysates (WC), cytosol and membrane fractions (CM) and cytoskeleton fractions (CSK) of non-treated (NT) and 10 μM Chloroquine (CQ) (24 h) treated HEK-KWGF cells. All three cytoskeleton components were included as well as a ponceau staining, which was used as loading control. Cleaved K_IR_2.1-GFP, indicated with an asterisk, is only present after CQ treatment. Fl, full-length K_IR_2.1-GFP. **(B)** Quantified results of the CQ induced induction of K_IR_2.1-GFP expression (*n* = 6). One-way ANOVA was used to calculate significance. **(C)** Quantified results of cleaved/fl K_IR_2.1-GFP ratios in all cell fractions (*n* = 6). One-way ANOVA was used to calculate significance.

We previously observed that CQ treatment resulted in increased K_IR_2.1 expression levels in HEK293 cells (Jansen et al., 2008; Varkevisser et al., 2013). In view of the recent work of Kang et al. (2020) that concluded that CQ affects cytoskeleton stability in podocytes, we assessed a potential effect of CQ on K_IR_2.1 interaction with the cytoskeleton. Therefore, HEK-KWGF cells were treated with 10 μM CQ for 24 h. Subsequently, whole cell (WC), cytoplasm + membrane (CM) and cytoskeleton (CSK) fractions were prepared and analyzed by western blot (Figure 5A). CQ treatment appeared to increase K_IR_2.1-GFP expression levels in all fractions, however, the strongest increase was found in the cytoskeleton fraction (2.6 ± 0.9 fold, 2.2 ± 0.5 and 103 ± 90 fold for WC, CM and CSK fractions, respectively) (Figure 5B). The fractionation procedure, at 4°C, resulted in isolation of cytoskeletons consisting mainly of actin and intermediate filaments, but no tubulin (Figure 5A). Upon CQ, a K_IR_2.1-GFP degradation product that results from N-terminal cleavage in the K_IR_2.1 protein (Varkevisser et al., 2013), became apparent in all three fractions (Figure 5A, marked with *). This cleaved product associated with the CSK as well, even reaching higher cleaved/full length ratios than in WC and CM fractions, which is indicative for a certain amount of enrichment (Figure 5C).

**FIGURE 6:**
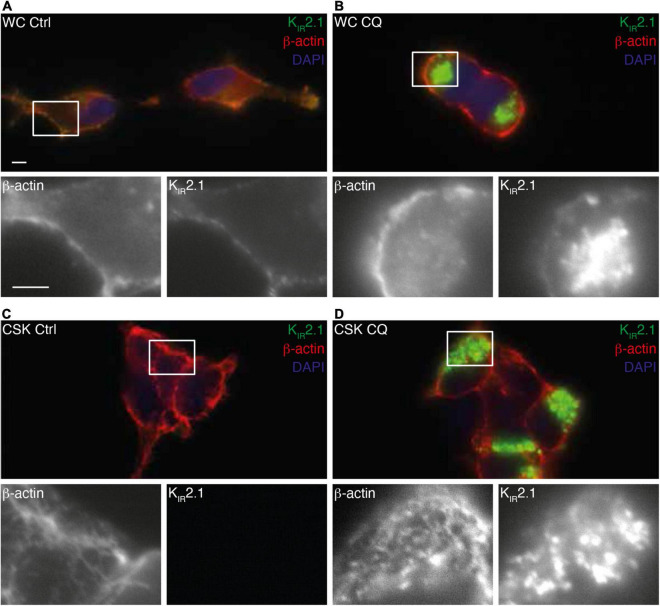
Interaction of K_IR_2.1-GFP with the cell cytoskeleton following Chloroquine treatment. Co-staining of K_IR_2.1-GFP (green) and β-actin in non-treated whole cell (WC) **(A,B)** or cytoskeleton isolated (CSK) **(C,D)** HEK-KWGF cells. Cells were either non-treated (Ctrl) **(A,C)** or treated with 10 μM CQ for 24 h **(B,D)**. Single staining patterns of the boxed areas are shown below each picture. Scale bar represents 5 μm.

Since the K_IR_2.1-GFP degradation product is not found at the plasma membrane (Varkevisser et al., 2013), our findings suggest interaction of non-plasma membrane associated K_IR_2.1-GFP with the cytoskeleton following CQ treatment. Fluorescent microscopy on whole cells and isolated cytoskeletons (Figure 6) demonstrates intracellular accumulation of K_IR_2.1-GFP upon CQ treatment (Figures 6A,B) as shown before (Jansen et al., 2008; Varkevisser et al., 2013). Indeed, these intracellular K_IR_2.1-GFP loaded structures are strongly associated with isolated cytoskeleton (Figure 6D). Close inspection demonstrates that K_IR_2.1-GFP signals in at least some of the intracellular vesicles are surrounded by and/or in close vicinity of actin, while all vesicles are in subcellular areas with dense actin signals (Figure 6D, inset).

## Discussion

Using a transient transfection system of the photoconvertible fluorescent protein Dendra2, we were able to optically quantify the differences in the dynamics of “old” and “new” proteins on the plasma membrane based on their red and green fluorescence intensity, respectively. Furthermore, we showed how disruption of the cytoskeleton slowed down forward and backward trafficking of the K_IR_2.1 channel protein. The intensity of green and red fluorescence was fitted by a double exponential function to separate the fast (τ1) and slow (τ2) component of fluorescent changes by time. The fast component of green recovery and red decay did not change significantly by the treatment with either drug, suggesting that τ1 for the fluorescence changes was due to the planar movement of the Dendra2-tagged proteins along the plasma membrane and therefore out of the ROI for fluorescent measurement. In control cells, we observed that the slow time constants for forward trafficking and backward trafficking were in the same order of magnitude (13.1 *vs*. 20.2 min, respectively), as could be expected under conditions of homeostasis at 48 h after transfection. Cytoskeletal modification affected the slow component of forward and backward trafficking of K_IR_2.1 channels. The τ2 for green fluorescence recovery prolonged significantly through Nocodazole and Cytochalasin B, indicating that the forward trafficking of K_IR_2.1 channels strongly depends on the tubulin and actin network. Although the differences were not statistically significant, the τ2 for red fluorescent decay in the cells treated with Nocodazole and Cytochalasin B was also prolonged compared to control cells. Loewen et al. (2009) speculated that K_IR_2.1 forward trafficking was particularly sensitive for Nocodazole-mediated tubulin disruption in contrast to some other cardiac potassium channels. Indeed, our time constant for forward trafficking was more strongly affected by Nocodazole compared to backward trafficking (approximately 12 *vs.* 3-fold), consistent with a net decrease in K_IR_2.1 ion channels at the plasma membrane. Furthermore, disruption of the actin skeleton also affected forward trafficking more potently than backward trafficking (approximately 10 *vs*. 3-fold). Consequently, we show that 24 h Cytochalasin B treatment decreased functional K_IR_2.1 channels at the plasma membrane as was evident from a decrease in *I*_*KIR*2_._1_. However, we cannot exclude that channel activation, rather than expression, also contributes to a decrease in *I*_*KIR*2_._1_. For example, Cytochalasin D has been shown to affect enzymes in the phospatidyl-4,5-biphosphate (PIP_2_) metabolic pathways (Vasilev et al., 2021). PIP_2_ is an important factor for K_IR_2.1 activity (Huang et al., 1998). Inhibition of dynamin motor proteins, as performed here through treatment with Dyngo-4a, was more potent on backward than on forward trafficking (approx. 4.3 *vs.* 2.5-fold). This latter finding is consistent with earlier work from Loewen et al. (2009) and Varkevisser et al. (2013) who used other molecular and pharmacological tools for dynamin inhibition, resulting in an increased ectopic K_IR_2.1 current in HEK293 cells.

The cytoskeleton contributes not only to the transport of membrane proteins but also to many other cellular biological processes including maintenance of cell shape, cell migration, cell division, endocytosis, and cell polarization (e.g., Pollard and Cooper, 2009; Kopf et al., 2020). Therefore, when using pharmacological approaches for cytoskeletal modulation as in our study, it should be considered that various cell biological processes are also affected by the drug mediated cytoskeleton modifications, as this altered cell biology may have indirect effects on ion channel trafficking too.

Obviously, K_IR_2.1 trafficking depends on many other interacting proteins, of which a number are known already like clathrin and AP1 (e.g., Tong et al., 2001; Ma et al., 2011). Furthermore, channel complexes, like K_IR_2.1 and Nav1.5 may actively cooperate to regulate their trafficking (Ponce-Balbuena et al., 2018). It can be assumed that the cytoskeleton may also have a role in steering these interacting proteins in the processes of K_IR_2.1 trafficking.

In earlier studies we demonstrated that (1) CQ induces dose- and time-dependent increased expression levels of non-tagged or tagged K_IR_2.1 in four different cell lines, which was accompanied with intracellular accumulation of full-length and K_IR_2.1 degradation products in lysosomes, (2) that CQ could counteract drug-induced defects in K_IR_2.1 expression, and (3) that CQ induced increased K_IR_2.1 dependent inward rectifier current (Jansen et al., 2008; Nalos et al., 2011; Varkevisser et al., 2013; Ji et al., 2017; Korte et al., 2017). However, a link to the cytoskeleton was not made earlier, which led us to perform cell fractionation assays and K_IR_2.1 imaging on isolated cytoskeletons. Our cell fractionation method was based on established protocols in which 0.5% non-ionic detergent Triton X-100 was combined with tubulin depolarization at low temperature. This concentration of Triton X-100 extracts all non-cytoskeleton bound proteins and phospholipids from the cytoskeleton (Van Bergen en Henegouwen et al., 1989), whereas extraction at low temperature (4°C) (Van Bergen en Henegouwen et al., 1992) yields a cytoskeleton fraction nearly devoid of tubulin, as shown here also in Figure 5A. Therefore, K_IR_2.1 interaction with the cytoskeleton shown here most likely represents K_IR_2.1 interaction with the actin and/or intermediate filament system. Given the proven interaction of K_IR_2.1 with actin binding proteins (Sampson et al., 2003), we may speculate that K_IR_2.1-actin interaction is dominant in the isolated CSK fraction. Besides K_IR_2.1 trafficking being dependent on a functional actin network, as shown here, it was demonstrated recently that membrane-localized functional K_IR_2.1 channels modulate actin filament dynamics (Wu et al., 2020). These authors suggest a role for K_IR_2.1 channels on cellular morphology, migration and adhesion, *via* the actin filament system.

The interaction of lysosomal localized K_IR_2.1 channel proteins with the actin/intermediate filament system must be considered in view of the crucial role of these cytoskeletal systems in late-endosome and lysosome organelle transport within the cell (Styers et al., 2004; Bonifacino and Neefjes, 2017), and therefore it may not be surprising that the late-endosome/lysosome cargo also interacts with these cytoskeletal elements, especially when the cargo itself is an actin binding protein such as K_IR_2.1. Since tubulin filaments depolarize at low temperatures as used in our fractionation and immunofluorescent microscopy assays (Van Bergen en Henegouwen et al., 1992), we cannot rule out that late-endosomal/lysosomal localized K_IR_2.1 channels also associate with tubulin filaments in intact cells.

In the failing heart, cardiomyocytes have less K_IR_2.1 expression compared to healthy counterparts (Borlak and Thum, 2003), whereas K_IR_2.1 expression is increased in cardiac fibroblasts in profibrillatory atrial tissue (Qi et al., 2015). Although changes in K_IR_2.1 channels and *I*_*K*1_ in failing hearts have been explained by changes in mRNA and channel proteins so far, this study emphasizes the need to consider the membrane transport of channels *via* the cytoskeleton too. It has been reported that cytoskeletal remodeling occurs in the failing hearts. In particular, tubulin, a component of microtubules, has been found to display an increased expression and excessive polymerization in pressure-loaded animal models (Stones et al., 2012) and human failing heart muscle in dilated cardiomyopathy (Heling et al., 2000). Recent studies have demonstrated that detyrosination, which inhibits excessive polymerization of tubulin, might improve contractile and diastolic function of cardiomyocytes in the failing myocardium (Chen et al., 2018). Microtubule-associated proteins (MAPs), such as tau, MAP4, and EB-1, are essential for the transport of membrane proteins, and the balance between microtubule polymerization and MAP binding determines membrane transport. The K_IR_2.1 protein interacts with actin through filamin-A, an actin-binding protein, but how K_IR_2.1 might interact with tubulin filaments is not yet known. Further studies are needed to understand how microtubule remodeling in the failing heart affects membrane trafficking of channel proteins, and live imaging using the Dendra2 protein used in this study might be useful.

## Limitations

Our work comes with several limitations. We studied the role of the cytoskeleton in K_IR_2.1-Dendra2 trafficking in human cell lines, rather than in adult ventricular human cardiomyocytes, for several practical and scientific reasons. Adult human ventricular cardiomyocytes are scarcely available for basic research. Furthermore, our approach requires cells to become transfected with K_IR_2.1-Dendra2 and treated with drugs for considerable timeframes, which would require culturing of the isolated cells. However, this would result in dedifferentiation of the rod-shaped cells (e.g., Bugaisky and Zak, 1989; De Boer et al., 2006a), most likely affecting also the cytoskeleton structure, and thus would still not mimic human adult ventricular cardiomyocytes completely. We considered human (induced pluripotent) stem cell derived cardiomyocytes, but this approach would suffer from their immature character (e.g., absence of an anisotropic phenotype, absence of T-tubuli, spontaneous active action potential formation, depolarized membrane potential, immature calcium handling). This limitation could be largely overcome by generating K_IR_2.1-Dendra2 transgenic animal models and isolating the adult ventricular cardiomyocytes. Of particular interest would be crossings of such a transgenic model with animal models of cardiac disease, or interventions resulting in for instance in heart failure, like ischemia/reperfusion or chronic AV block. Therefore, our technologies and findings may be an important first step toward better understanding of the cytoskeleton’s role in K_IR_2.1 trafficking in the healthy and diseased heart.

TIRF microscopy is currently the best tool to separate fluorescent information from the plasma membrane from intracellular fluorescence. However, the combination of the available TIRF setup with the technical challenging and time-restricted processes of photoconversion and dual-channel live imaging prevented the use of this technology. We decided to perform imaging by better established and validated confocal laser-scanning microscopy instead. By using the flat HeLa cells, rather than more rounded HEK293 cells for imaging, we were able to photoconvert only the proteins on the cell membrane as far as possible.

## Data Availability Statement

The original contributions presented in the study are included in the article/Supplementary Material, further inquiries can be directed to the corresponding author.

## Author Contributions

EL, VL, WH, WK, and HT performed research. MH, EL, VL, WH, WK, and HT analysed the results. MH and HT designed the study and wrote the manuscript. All authors reviewed and approved the final manuscript.

## Conflict of Interest

The authors declare that the research was conducted in the absence of any commercial or financial relationships that could be construed as a potential conflict of interest.

## Publisher’s Note

All claims expressed in this article are solely those of the authors and do not necessarily represent those of their affiliated organizations, or those of the publisher, the editors and the reviewers. Any product that may be evaluated in this article, or claim that may be made by its manufacturer, is not guaranteed or endorsed by the publisher.
