# Supplementary material for: Quantitative Analysis of the Cytoskeleton’s Role in Inward Rectifier KIR2.1 Forward and Backward Trafficking
Source: Front Physiol. 2022 Jan 25;12:812572. doi: 10.3389/fphys.2021.812572 (PMC8821923; doi:10.3389/fphys.2021.812572)
Supplement: Supplementary file 1 [file Data_Sheet_1.PDF]

Supplementary material

Assessment of intracellular accumulation of K_{IR}2.1-Dendra2 in fluorescent images

Methods

Cells were scored in three different classes: "no accumulation" when accumulation was hardly observed, "vesicular accumulation" when accumulation was observed in vesicles with a diameter of 10 µm or less, and "massive accumulation" when aggregation was observed with a diameter of 10 µm or more. In total, 17 control, 19 Dyngo4a, 20 Nocodazole and 20 Cytochalasin B treated cells, from at least five independent dishes used for photoconversion experiments were evaluated.

Results

Treatment with Dyngo4a reduced the intracellular accumulation of K_{IR}2.1-Dendra2. Cytoskeletal disruption by Nocodazole or Cytochalasin B increased the intracellular accumulation of K_{IR}2.1-Dendra2. Massive intracellular accumulation of Kir2.1-Dendra2 was observed in particular following impairment of the actin filament system.

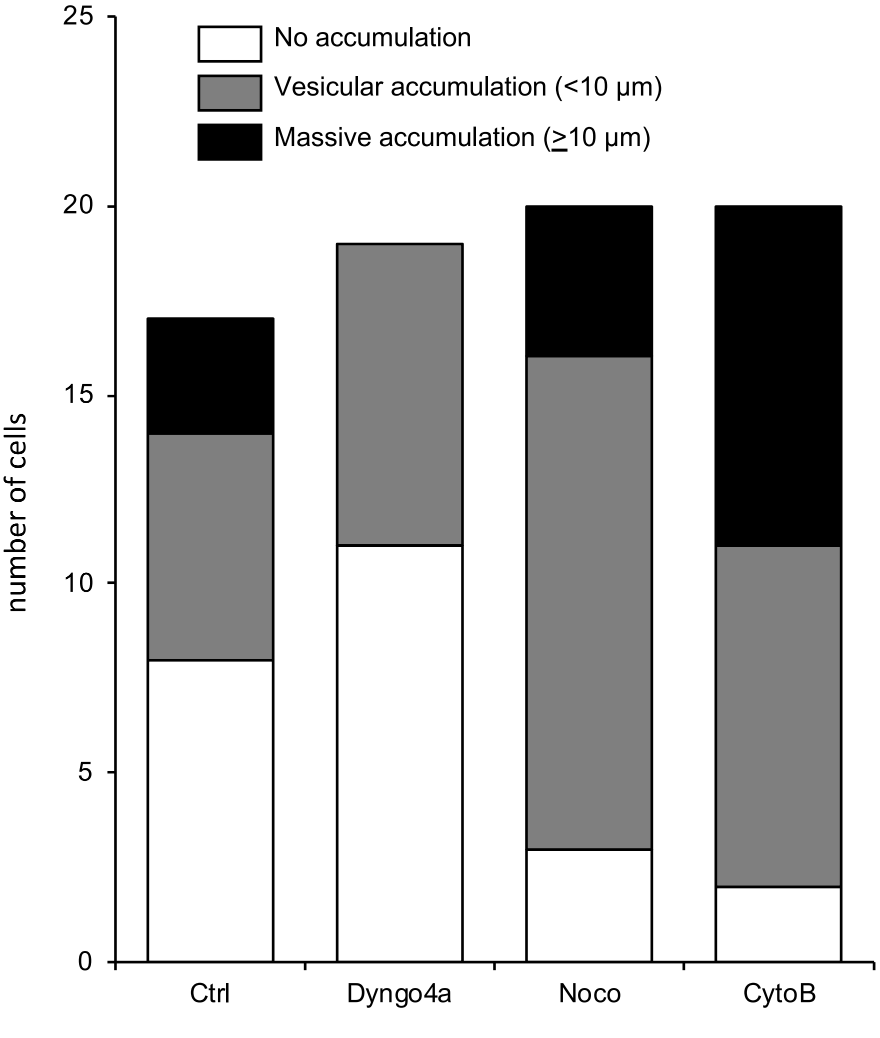

Figure S1 Accumulation of K_{IR}2.1-Dendra2 in individual cells treated with Dyngo4a, Nocodazole (Noco) or Cytochalasin B (CytoB). Cells were classified as displaying no or minimal intracellular accumulation (white), vesicular accumulation of less than 10 µm (grey) or massive accumulations of larger than 10 µm (black). Number of cells are indicated.
